# Case Report: Recurrent pathogenic mutation c.110G>A in *DHDDS* gene

**DOI:** 10.3389/fnins.2026.1801725

**Published:** 2026-05-11

**Authors:** Ci Liu, Qiaoyu Ye, Jie Li, Siming Bu, Chenlu Ge, Xiangnan Wang, Moyuan Quan, Liang Wang

**Affiliations:** 1 Prenatal Diagnosis Center, the Second Hospital of Hebei Medical University, Shijiazhuang, Hebei, China; 2 Key Laboratory of Clinical Neurology, Ministry of Education, Hebei Medical University, Shijiazhuang, Hebei, China; 3 Department of Neurology, The Second Hospital of Hebei Medical University, Shijiazhuang, Hebei, China; 4 Key Neurological Laboratory of Hebei Province, Shijiazhuang, Hebei, China; 5 Department of Emergency, Harrison International Peace Hospital Affiliated to Hebei Medical University, Hengshui, Hebei, China; 6 Department of Clinical Medicine, Hebei Medical University, Shijiazhuang, Hebei, China

**Keywords:** ataxia, DHDDS, epilepsy, myoclonus, whole exome sequencing (WES)

## Abstract

Recent studies have demonstrated the close association of mutations in the dehydrodolichyl diphosphate synthase (*DHDDS)* gene with neurodevelopmental disorders and the onset of epilepsy. This report describes a female patient harboring a *de novo* heterozygous variant c.110G>A (p.Arg37His) in the *DHDDS* gene, characterized by childhood-onset myoclonus-like movement disorder (at age 6) and late-onset epilepsy (at age 17). The movement disorder was remarkably improved through the levetiracetam+ clonazepam+ haloperidol triple therapy, and epileptic seizures were also effectively controlled. A retrospective analysis of 59 epilepsy patients with *DHDDS* gene variants revealed significant clinical heterogeneity in disease phenotypes caused by *DHDDS* mutations. Epilepsy was identified as the predominant symptom, commonly accompanied by movement disorders and varying degrees of intellectual disability. Furthermore, while pathogenic mutations in *DHDDS* tend to be relatively clustered, no definitive genotype-phenotype correlation has been established. This study highlights the clinical manifestations, imaging features, treatment experiences, and genetic testing results through case reports and literature review, thereby providing crucial references for the clinical diagnosis, treatment, and further research of such diseases.

## Introduction

Epilepsy ranks as the second most prevalent neurological disorder globally, and genetic factors are considered the primary cause in up to 80% of patients (Zhang et al., 2024). Its genetic mechanisms are complex, involving abnormalities in genes across multiple pathways, including ion channels, synaptic transmission, and metabolic regulation. Driven by rapid advances in genomic technologies, significant progress has been made in identifying pathogenic genes responsible for monogenic epilepsies. Currently, over 1,500 associated pathogenic genes have been identified of which approximately 10% are genes with epilepsy as the core symptom and are strongly implicated in epileptogenesis, while approximately 24% are associated with both neurodevelopmental disorders (the primary symptomatic manifestation) and epilepsy. In recent years, mutations in the dehydrodolichyl diphosphate synthase (*DHDDS)* genes have been confirmed to be

associated with neurodevelopmental disorders and the onset of epilepsy. Biallelic mutations in the *DHDDS* gene are related to various autosomal recessive neurodevelopmental and multisystem disorders, with some cases presenting retinopathy (Jiao et al., 2022; Lam et al., 2014; Sabry et al., 2016). In contrast, heterozygous mutations predominantly cause the autosomal dominant developmental and epileptic encephalopathy with or without movement disorders (DEDSM, OMIM: 617836). The characteristic phenotype of DEDSM includes infantile-onset seizures (such as myoclonus, tonic-clonic seizures), progressive neurodevelopmental delay or regression, and movement disorders (such as ataxia, tremor, and dystonia). It is typically caused by *de novo* mutations, and inheritance from parents with mild phenotypes is rarely reported (Dong et al., 2023; Galosi et al., 2022; Wood et al., 2021). In this study, clinical phenotypic and molecular genetic analyses were performed on one patient with *DHDDS*-related movement disorder. By reviewing the patient's diagnostic and treatment experience and simultaneously performing a literature review of the clinical and genetic characteristics of previously reported patients with *DHDDS* gene variants, we aim to improve clinical understanding of this rare disease and provide patients with personalized diagnosis and treatment.

## Medical records and results

*Case description:* A 20-year-old female patient presented with involuntary movements of the extremities and face for 14 years, and intermittent convulsive seizures for 3 years. Fourteen years ago, she developed involuntary movements of the thumbs and index fingers of both hands with no identifiable trigger, manifested as non-rhythmic tremors. These symptoms gradually involved the cheeks, tongue, jaw, neck, and extremities, predominantly in the distal upper limbs, impairing fine motor skills such as grasping objects. The symptoms exacerbated during emotional stress, accompanied by intellectual developmental delay, dysarthria, and inability to complete primary school education. Seven years ago, she developed difficulty in walking with aggravated tremors in both lower limbs, presenting as inability to walk independently and requiring assistance from family members. Additionally, intermittent weakness of both lower limbs occurred during walking, without accompanying convulsions or numbness of the extremities. The patient presented to a local hospital, where a working diagnosis of dopa-responsive dystopia (DRD) and spinocerebellar ataxia (SCA) was made. Genetic testing, specifically SCA fragment analysis, revealed no significant abnormalities. Levodopa/benserazide was administered orally, but there was no significant improvement in symptoms. Three years ago, the patient's symptoms of involuntary movements involving the face and extremities significantly worsened, with a further decline in the ability to perform activities of daily living, requiring assistance from others for daily activities such as eating. Additionally, generalized tonic-clonic seizures (GTCS) occurred, manifesting as upward gaze of both eyes, salivation, limb rigidity, and loss of consciousness, lasting several minutes and occurring several times a year. In October 2024, the patient was admitted to the hospital for treatment due to another epileptic seizure. The patient's parents are non-consanguineous, with no family history of similar diseases.

## Physical examination

The patient had clear consciousness, with decreased responsiveness and orientation. Dysarthria was present, characterized by syllable repetition and articulatory interruption. Myoclonic involuntary movements were observed in the face, tongue, and mandible, along with neck tremor. Muscle strength of all extremities was Grade 5. Increased muscle tone was noted in all extremities, with myoclonic involuntary movements involving all extremities and coarse postural tremor of both upper extremities. Tendon reflexes of both upper extremities were normal, while those of both lower extremities were hyperactive. The Babinski sign was negative bilaterally. Sensory system examination revealed no obvious abnormalities. The finger-to-nose test revealed bilateral dysmetria, and rapid alternating movements were impaired (dysdiadochokinesia). The patient was uncooperative with the heel-to-shin test and Romberg test. Gait was characterized by slowness with a widened base, and bilateral foot inversion; walking could induce coarse, myoclonus-like involuntary movements of both lower extremities.

## Ancillary tests

**Figure 1:**
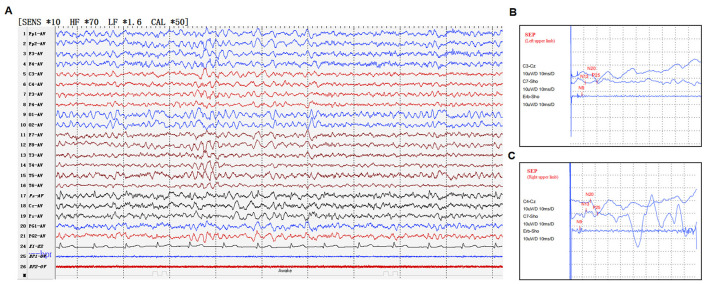
**(A)** Electroencephalogram (EEG) and **(B, C)** Somatosensory evoked potential (SEP) of the patient.

Genetic testing for dynamic mutations in SCA genes performed 7 years ago yielded unremarkable results. During the hospitalization in 2024, brain MRI scan was performed, which revealed no abnormal findings. Long-term video-electroencephalography (EEG; November 28th, 2024) demonstrated a normal electroencephalogram. Video electroencephalogram (VEEG; December 20th, 2024) showed moderately abnormal background activity characterized by moderate slowing of occipital rhythms and generalized θ wave discharges during wakefulness. Somatosensory evoked potentials (SSEPs) revealed normal latencies and amplitudes in the cortical, spinal, and peripheral segments bilaterally. No giant potentials were observed bilaterally at N20 (Figure 1). Ceruloplasmin level (November 28th, 2024) was 0.34 g/L (reference range typically 0.20–0.50 g/L for adults). Autoantibody screen + anticardiolipin antibodies (IgG, IgM) + Antineutrophil cytoplasmic antibodies (ANCA; November 28th, 2024) revealed no significant abnormalities.

## Preliminary diagnosis: 1. Epilepsy; 2. Myoclonus

### Clinical treatment

Levetiracetam was administered for antiepileptic therapy, with no recurrence of seizures observed. Clonazepam and haloperidol were given to improve involuntary movements, which, along with difficulty in walking of the patient, were significantly alleviated.

### Genetic testing and analysis

**Figure 2:**
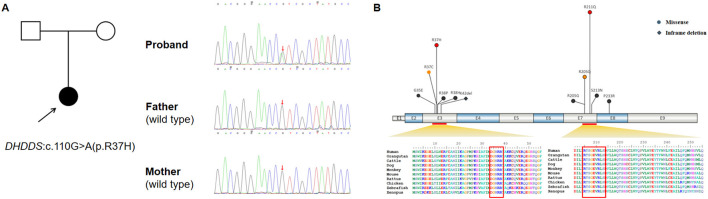
**(A)** Clinical information and variant information. Pedigree of the family and confirmation of heterozygous *DHDDS* c.110G>A (p.R37H) by Sanger sequencing. **(B)** Evolutionary conservation of *DHDDS* variants (The red-boxed regions demarcate amino acid residues exhibiting extreme evolutionary conservation across vertebrate orthologs, wherein reported pathogenic variants are clustered).

Trio-based whole-exome sequencing (WES) was performed on the proband and her parents, revealing that the proband harbored a heterozygous missense mutation c.110G>A in exon 3 of the *DHDDS* gene. Neither parent carried this mutation, indicating it is a *de novo* mutation (Figure 2A). The c.110G>A variant, one of the most common missense mutations in the *DHDDS* gene, has been reported in at least 10 patients with *DHDDS*-related neurodevelopmental disorders, all of which are *de novo* mutations (Galosi et al., 2022; Hamdan et al., 2017; Jiao et al., 2022). The c.110G>A mutation results in the replacement of arginine with histidine and is located at a highly evolutionarily conserved site, which has not been identified in the 1,000 Genome, ExAC, and Genome Aggregation Database (gnomAD). In addition, existing functional studies have shown that the c.110G>A variant leads to a significant reduction in aspartase activity (Bar-El et al., 2020). Moreover, Provean, Polyphen2, Sift, Mutationtaster, and REVEL software predictions indicate that the protein structure at this mutation site is likely deleterious. According to the The American College of Medical Genetics and Genomics (ACMG) Standards and Guidelines for the Classification of Genetic Variants (Richards et al., 2015), this variant meets the pathogenic criteria (PS2_VeryStrong, PM2_Supporting, PS3_Supporting, PP3).

## Revised diagnosis: 1. *DHDDS*-related movement disorder; 2. Epilepsy

### Follow-up

After discharge, the patient continued to take oral medications: levetiracetam 0.75 g twice daily, clonazepam 0.25 mg twice daily, Haloperidol 0.5 mg once nightly, and trihexyphenidyl hydrochloride 2 mg twice daily. Regular outpatient follow-up visits and active rehabilitation therapy were maintained. Recent follow-up of the patient showed a reduction in the amplitude of involuntary movements of the head and face, and a decrease in the tremor of the limbs. The patient could perform activities such as grasping, feed independently, and walk independently, with increased walking speed and no falls. No epileptic seizures occurred. The patient's social communication skills showed improvement, as evidenced by expanded vocabulary, increased eye contact, and more frequent social interactions (e.g., sharing and commenting).

## Literature review results

Using “*DHDDS* gene”, “*DHDDS* variants,” and “dehydrodolichol diphosphate synthase” as the keywords, we searched the PubMed database, Web of Science, CNKI, and Wanfang Database for Chinese and English literature from their inception to April 2025. There were 14 articles in English and 1 in Chinese that met the retrieval criteria; there were a total of 59 patients, including 1 from this study. A total of 11 *DHDDS* mutations have been reported (Álvarez-Mora et al., 2022; Courage et al., 2021; Dong et al., 2023; Fernández-Marmiesse et al., 2019; Galosi et al., 2022; Hamdan et al., 2017; Jiao et al., 2022; Josephs et al., 2019; Kim J. et al., 2021; Kim et al., 2022; Kim S. et al., 2021; Liu et al., 2024; Sedlackova et al., 2024; Ware et al., 2019; Wood et al., 2021; Table 1), including 10 missense mutations and 1 in-frame deletion, the vast majority of which are *de novo* mutations (54/59). Among these variants, c.632G>A and c.110G>A are the two most common recurrent pathogenic variants, accounting for 37.2% (22/59) and 23.7% (14/59) respectively. All mutation sites are significantly enriched in two regions: Five variants (c.104G>A, c.109C>T, c.110G>A, c.113G>A, and c.113G>C) are located in the catalytic domain of exon 3 (amino acid positions D34 to R38), and four variants (c.613C>G, c.614G>A, c.632G>A, and c.638G>A) are located in the isopentenyl diphosphate binding site (IPP) of exon 7. Both regions are highly conserved during evolution (Galosi et al., 2022; Jiao et al., 2022; Figure 2B). Previous studies have shown that R37H and R211Q mutations directly interfere with substrate binding, leading to a significant reduction in catalytic activity (Galosi et al., 2022).

**Table 1:** Genetic features and ACMG pathogenicity classification of reported *DHDDS* variations, with ClinVar-curated variants highlighted by asterisks.

*DHDDS* variant	Exon	Variant type	Number of cases	ACMG pathogenicity classification
c.104G>A, p.G35E^*^	3	Missense variant	2	Pathogenic:PS2 + PS3_P + PP3_S + PM1 + PM2_P
c.109C>T, p.R37C^*^	3	Missense variant	7	Pathogenic:PS2_VS + PS3_P + PP3 + PM1 + PM2_P
c.110G>A, p.R37H^*^	3	Missense variant	14	Pathogenic:PS2_VS + PS3_P + PP3 + PM1 + PM2_P
c.113G>A, p.R38H^*^	3	Missense variant	1	Pathogenic:PS2_M + PS3_P + PP3_M + PM1 + PM2_P
c.113G>C, p.R38P	3	Missense variant	1	Likely pathogenic:PS2_M + PP3_M + PM1 + PM2_P
c.124_126del, p.K42del	3	Inframe deletion	1	Uncertain significance:PS2_M + PM4_P + PM2_P
c.613C>G, p.R205G	7	Missense variant	1	Uncertain significance:PS2_M + PP3_M + PM2_P
c.614G>A, p.R205Q^*^	7	Missense variant	6	Pathogenic:PS4_P + PS2 + PS3_P + PP3_M + PP1 + PM2_P
c.632G>A, p.R211Q^*^	7	Missense variant	22	Pathogenic:PS3_VS + PS3_P + PP3_M + PM2_P
c.638G>A, p.S213N^*^	7	Missense variant	2	Likely pathogenic:PS2 + PS3_P + PP3 + PM2_P
c.698C>G, p.P233R^*^	8	Missense variant	1	Likely pathogenic:PS2_M + PS3_P + PP3_M + PM2_P

VS, very strong; S, strong; M, moderate; P, supporting. ^*^Variant curated by ClinVar.

A pooled analysis of the 59-patient cohort revealed that although *DHDDS* mutation hotspots were clustered at the R37, R38, R205, and R211 loci (all arginine residues), no clear correlation was observed between genotype and clinical phenotype. These variants could not predict disease severity in terms of cognitive function, epilepsy, or motor dysfunction phenotypes. Among them, the two most common variants (p.R37H and p.R211Q) showed no significant differences in age of onset, cognitive ability, motor dysfunction, and disease course. For instance, another 13 patients carrying the same R37H variant as the cases in this study exhibited high heterogeneity: Age of onset ranged from infancy to adolescence, and neurodevelopmental impairment was distributed across a spectrum from severe to mild cognitive deficits, suggesting that a single variant could not predict clinical manifestations or course of disease (Table 2). However, the disease course of patients with lower degrees of cognitive impairment might be characterized by later onset, milder epilepsy, and milder motor dysfunction, independent of specific variants.

**Table 2:** Summary of the clinical features of individuals with DHDDS mutations.

No.	Patient ID	Sex	Variant (NM_205861.3)	Inheritance	Cognition	Movement disorder	Brain MRI	Seizure onset	Reference
1	15	F	c.104G>A	de novo	Moderate ID	Tremor, chorea, myoclonus, dystonia, and ataxia	Normal	3 years	2
2	21	M	c.104G>A	de novo	Moderate ID	Ataxia, tremor, chorea, and rigidity	Corpus callosum thickening	11 months	2
3	7	M	c.109C>T	de novo	Severe ID	Tremor, myoclonus, and chorea	Normal	11 months	2
4	8	F	c.109C>T	de novo	Moderate ID	Tremor, ataxia, myoclonus, and dystonia	Normal	2 years	2
5	13	F	c.109C>T	de novo	Severe ID	Myoclonus, ataxia, and stereotypies	Normal	Childhood	2
6	14	F	c.109C>T	de novo	Severe ID	Myoclonus, ataxia, and stereotypies	Normal	Childhood	2
7	1	F	c.109C>T	de novo	Severe ID	Tremor, ataxia	Normal	1 year	3
8	7	/	c.109C>T	de novo	Severe ID	Tremor, ataxia, and hypertonia	Normal	10 months	3
9	/	F	c.109C>T	de novo	Moderate ID	Generalized myoclonus, ataxia	/	No seizure	4
10	/	F	c.110G>A	de novo	Moderate ID	Tremor, myoclonus, dystonia, and ataxia	Normal	17 years	This study
11	3	M	c.110G>A	de novo	Mild ID	Tremor, ataxia	Normal	1 year	2
12	10	M	c.110G>A	de novo	Severe ID	Stereotypies	Normal	4 years	2
13	12	M	c.110G>A	de novo	Mild ID	Ataxia, myoclonus, tremor, parkinsonism, and spasticity	Normal	19 years	2
14	25	M	c.110G>A	de novo	Moderate ID	Ataxia	Normal	8months	2
15	4	F	c.110G>A	de novo	Severe ID	Tremor, hypertonia	Atypical signals in bilateral frontal and parietal white matter	8months	3
16	5	M	c.110G>A	de novo	Severe ID	/	Normal	13 months	3
17	6	M	c.110G>A	de novo	Moderate ID	Tremor, ataxia, and hypertonia	Hypomyelination of white matter, Widened subarachnoid space	15 months	3
18	9	M	c.110G>A	de novo	Severe ID	/	Atypical signals in bilateral frontal and parietal white matter	4 years	3
19	indvSG	F	c.110G>A	de novo	Severe ID	Hypotonia	Normal	18 months	5
20	HSJ0762	M	c.110G>A	de novo	GDD	Hypotonia, tremor, wide based gate, and ataxia	Normal	1 year	5
21	6	M	c.110G>A	de novo	Mild ID	Myoclonic, hypotonia, and ataxia	/	18 months	6
22	1197	M	c.110G>A	de novo	/	Tonic rigidity	/	5 years	7
23	Case study 2	M	c.110G>A	de novo	Moderate ID	/	/	5 years	8
24	proband (III-1)	M	c.113G>A	Maternally inherited	Mild ID	Intentional tremors, myoclonus	/	8 years	9
25	8	M	c.113G>C	de novo	Severe ID	Tremor, ataxia	Normal	4 years 3 months	3
26	24	F	c.124_126del	de novo	Severe ID	Tremor, myoclonus, and parkinsonism	Normal	7months	2
27	Case 1	F	c.613C>G	de novo	Delayed Intelligence and motor	Unsteady gait, motor regression, tremor, and hypotonia	/	3 years	16
					development				
28	19	F	c.614G>A	de novo	Mild ID	Tremor, ataxia, dystonia, and parkinsonism	Normal	1 year	2
29	2	F	c.614G>A	de novo	Severe ID	Tremor	Hypomyelination of white matter	2 years 6 months	3
30	Sibling 1	F	c.614G>A	Maternally inherited	GDD, severe learning disability	Tremor, myoclonus, ataxia, and orofacial dyskinesia	Normal	3 years	10
31	Sibling 2	F	c.614G>A	Maternally inherited	GDD, moderate learning disability	Hypotonia, myoclonus, tremor, orofacial dyskinesia, and ataxia	Normal	5 years	10
32	Their mother	F	c.614G>A	/	Mild learning disability	/	Normal	2 years	10
33	PME71	F	c.614G>A	/	Normal cognition	Ataxia, mild action myoclonus	/	17 years	11
34	1	M	c.632G>A	*de novo*	Severe ID	Tremor, ataxia, parkinsonism, and dystonia	Normal	5 years	2
35	2	F	c.632G>A	*de novo*	Moderate ID	Tremor, myoclonus	Normal	/	2
36	4	F	c.632G>A	*de novo*	Severe ID	Tremor, ataxia	/	10 months	2
37	5	M	c.632G>A	*de novo*	Moderate ID	Tremor, ataxia, and spasticity	Normal	/	2
38	6	M	c.632G>A	*de novo*	Severe ID	Tremor, myoclonus, parkinsonism, dystonia, and spasticity	Normal	10 years	2
39	11	F	c.632G>A	*de novo*	Severe ID	Ataxia, myoclonus, tremor, dystonia, and parkinsonism	Normal	11 months	2
40	16	F	c.632G>A	*de novo*	GDD	/	Normal	21 months	2
41	17	M	c.632G>A	*de novo*	/	Myoclonus, tremor, and ataxia	Normal	5 years	2
42	20	M	c.632G>A	*de novo*	Moderate ID	Tremor, ataxia, myoclonus, dystonia, and parkinsonism	Normal	6months	2
43	22	M	c.632G>A	*de novo*	Severe ID	Ataxia, myoclonus, and tremor	Normal	2 years	2
44	23	F	c.632G>A	*de novo*	Mild ID	Tremor	Normal	/	2
45	3	M	c.632G>A	*de novo*	Severe ID	Ataxia	/	8 months	3
46	10	F	c.632G>A	*de novo*	Severe ID	/	Rathke cyst	13 months	3
47	indvEF	F	c.632G>A	*de novo*	borderline IQ	Hypotonia, tremor, and ataxia	Norma, Chiari I malformation	4 years	5
48	MDB31882	M	c.632G>A	*de novo*	Severe ID	Tremor, facial myokimia, bradykinesia, hypomimia, rigidity, freezing and impaired postural reactions	Normal	6–9 years	5
49	indvNCJ	F	c.632G>A	*de novo*	Moderate-Severe ID	Ataxia, myoclonus, tremor, and dystonia	Normal	7 years	5
50	PME3	M	c.632G>A	*de novo*	Moderate ID	Myoclonus, mild ataxia	/	/	11
51	37	/	c.632G>A	*de novo*	/	Dystonia	/	/	12
52	308	/	c.632G>A	*de novo*	/	Tremor	/	/	12
53	Patient_11	F	c.632G>A	*de novo*	Severe ID	/	/	/	13
54	10	F	c.632G>A	*de novo*	Profound GDD	/	Normal	6 months	14
55	Case 2	F	c.632G>A	*de novo*	Delayed Intelligence and motor development	Muscle weakness	/	14 months	16
56	Case 3	M	c.632G>A	*de novo*	Delayed Intelligence and motor development	Hypotonia, Intentional tremors	/	3 years	16
57	18	M	c.638G>A	*de novo*	Severe ID	Tremor, ataxia, myoclonus, and parkinsonism	Normal	1 year	2
58	/	M	c.638G>A	*de novo* (assumed)	/	Eyelid myoclonus and tremulous head movements	Normal	14 years	15
59	9	M	c.698C>G	*de novo*	Mild ID	Chorea, myoclonus	Normal	10 years	2

## Discussion

To date, 11 *DHDDS* variants have been identified in 59 patients (including a case from the present study), resulting in various types of movement disorders, epilepsy, or other symptoms (Table 2). The disease is clinically heterogeneous, with epilepsy as the most prominent symptom. The age at first seizure ranges from 10 months to 19 years, and cases with onset during infancy and early childhood (0–6 years) accounted for 78% (40/51). Myoclonic seizures is the most common type of epilepsy, presenting as brief lightning-like jerks of the limbs or face, which can progress to myoclonic status epilepticus, followed in frequency by myoclonic absence seizures (accompanied by impaired consciousness and rhythmic myoclonus) and GTCS. Several patients have fever sensitivity, with a significant increase in seizure frequency during fever episodes. Movement disorders are predominantly postural/action tremor (35/51), affecting the upper limbs and head, and are aggravated by emotional excitement or light stimulation. Ataxia is characterized by unsteady gait and an increased risk of falling. Several cases present with dystonia (torsional postures of the limbs or face) and orofacial dyskinesia (such as involuntary tongue and mouth movements), and a minority develop superimposed parkinsonism-like symptoms (bradykinesia and muscle rigidity) with age (Galosi et al., 2022; Jiao et al., 2022).

In clinical practice, when tremor, ataxia and gait abnormalities are the predominant early manifestations of *DHDDS*-related disorders, they may be misdiagnosed as DRD or SCA, as occurred in this study. The following key points may aid in differential diagnosis: *DHDDS*-related disorders typically have an onset in infancy or childhood, characterized by a combined phenotype involving multisystem neurological impairment, including neurodevelopmental delay (global developmental delay/intellectual disability), epilepsy (mostly generalized seizures, with myoclonic seizures being common, though the onset age may vary), and mixed movement disorders (tremor, action myoclonus/cortical tremor, ataxia, dystonia, parkinsonism, etc.; Galosi et al., 2022; Jiao et al., 2022). Notably, such patients usually show a poor response to levodopa treatment, and neuroimaging findings are mostly non-specific. Cranial MRI is often normal or shows only non-specific changes (Galosi et al., 2022; Jiao et al., 2022). In contrast, the core feature of DRD include childhood-onset dystonia, often with significant diurnal fluctuation, and a dramatic and sustained response to low-dose levodopa; it is generally not associated with epilepsy or significant cognitive impairment. SCA typically follows a progressive course, and presents primarily with cerebellar syndrome, with MRI commonly revealing cerebellar (with or without brainstem) atrophy. Epilepsy and significant neurodevelopmental impairment are not the core features of the disease.

In this case, the patient showed no significant benefit from levodopa/benserazide treatment. The subsequent development of GTCS, coupled with an unremarkable brain MRI and long-standing neurodevelopmental impairment, further supports a diagnosis of *DHDDS*-related disorders over DRD/SCA. The final diagnosis was confirmed by trio-based WES, which identified a denovo pathogenic variant of *DHDDS* c.110G>A (p.Arg37His).

In the treatment of epilepsy, valproic acid has the highest control rate for myoclonic seizures, but monitoring of liver enzymes and blood lipids is required; levetiracetam is partially effective, and myoclonic status epilepticus requires urgent treatment with benzodiazepines. For movement disorders, trihexyphenidyl is the first-line therapy for improving tremor; as for refractory tremor, subthalamic nucleus deep brain stimulation (STN-DBS) may be considered (Hamdan et al., 2017; Jiao et al., 2022; Liu et al., 2024). It should be noted that carbamazepine/oxcarbazepine may exacerbate myoclonic seizures, and during the febrile period, a prophylactic increase of antiepileptic drug dosage is recommended (e.g., oral diazepam).

The *DHDDS* gene encodes the catalytic subunit (*DHDDS*) of cis-prenyltransferase (cis-PTase), which is involved in the biosynthesis of dolichol in the endoplasmic reticulum and its dependent protein glycosylation process, as well as the synthesis and modification of glycoproteins (Galosi et al., 2022). Protein N-glycosylation is a metabolic process involving the attachment of polysaccharides to asparagine or arginine residues of proteins, in which *DHDDS* plays an important role. Our study revealed that 7 out of 11 *DHDDS* genetic variants were characterized by the replacement of arginine by other amino acids (e.g., histidine, glutamine). We hypothesize that these amino acid changes may affect N-glycosylation substrate recognition through alterations in protein charge distribution or spatial conformational changes, but further functional validation is required.

In conclusion, we report the case of a patient with *DHDDS*-related movement disorder carrying a *de novo* mutation (c.110G>A) in the *DHDDS* gene. Compared with other patients harboring this variant, this case presented with myoclonus-like movement disorder and a later age of epilepsy onset. Despite limited treatment experience, the patient demonstrated a favorable response to antiepileptic drugs and medications for movement disorder, providing valuable clinical insights. Literature review revealed significant phenotypic heterogeneity in *DHDDS*-related diseases, with no clear genotype-phenotype correlation. This heterogeneity may be influenced by multiple factors, such as differences in molecular pathological mechanisms, modifier genes, and environmental factors, necessitating further investigation. In the future, accumulation of more cases and in-depth research will facilitate a better understanding of pathogenesis of *DHDDS*-related diseases and provide more scientific evidence for their diagnosis and management.

## Data Availability

The data presented in the study are deposited in the ClinVar repository, accession number SCV007540285.1.
