# Supplementary material for: Case Report: Recurrent pathogenic mutation c.110G>A in DHDDS gene
Source: Front Neurosci. 2026 May 11;20:1801725. doi: 10.3389/fnins.2026.1801725 (PMC13199298; doi:10.3389/fnins.2026.1801725)
Supplement: Supplementary file 1 [file Data_Sheet_1.pdf]

Supplementary Table S1: Clinical characteristics, treatment, and outcomes of previously reported patients with DHDDS c.110G>A (p.R37H).

Table2 No.	Patient ID	Seizure onset	Antiseizure medications (ASM)	Movement disorder*	Treatment for movement disorder	Outcome	Reference
11	3	1y	VPA ± LEV	Tremor, ataxia	NR	Seizure free for 2 years	2
12	10	4y	VPA	Stereotypies	NR	Well controlled	2
13	12	19y	VPA + PRM + LEV + CZP	Ataxia, myoclonus, tremor, parkinsonism, spasticity	NR	Well controlled, seizure free since 26 y	2
14	25	8mo	VPA + ZNS	Ataxia	NR	Refractory	2
15	4	8mo	VPA, OXC,LEV	Tremor, hypertonia	NR	No seizure for 18 months	3
16	5	13mo	VPA	NR	NR	No seizure for 2 years	3
17	6	15mo	VPA, LEV	Tremor, ataxia, hypertonia	NR	No seizure for 6 years	3
18	9	4y	OXC,VPA, LTG	NR	NR	No seizure for 6 months	3
19	indvSG	18mo	VPA,LTG,LEV,ETH	Hypotonia	NR	intractable seizures	5
20	HSJ0762	1y	LEV,VPA	Hypotonia, tremor, wide based gait, ataxia	NR	Seizure free for 1 year on VPA	5
21	6	18mo	NR	Myoclonic, hypotonia, ataxia	NR	NR	6
22	1197	5y	NR	Tonic rigidity	NR	NR	7
23	Case study 2	5y	NR	NR	NR	NR	8

Abbreviations: ASM, antiseizure medication; VPA, valproate; LEV, levetiracetam; LTG, lamotrigine; OXC, oxcarbazepine; ZNS, zonisamide; PRM, primidone; CZP, clonazepam; ETH, ethosuximide; NR, not reported.

*Movement disorders include tremor, ataxia, myoclonus, parkinsonism, stereotypies, hypotonia, hypertonia, and tonic rigidity as reported in the original studies.

Supplementary Table S2: Correspondence between cases listed in Table 2 and the originally reported cases in the literature (DHDDS c.110G>A, p.R37H).

Table 2 No.	Patient ID	First reported study	Location in original publication
10	Present case	This study	Case in this article
11	3	Galosi et al., 2022, Brain	Supplementary Table 1 (Patient 3)
12	10	Galosi et al., 2022, Brain	Supplementary Table 1 (Patient 10)
13	12	Galosi et al., 2022, Brain	Supplementary Table 1 (Patient 12)
14	25	Galosi et al., 2022, Brain	Supplementary Table 1 (Patient 25)
15	4	Jiao et al., 2022, Developmental Medicine & Child Neurology	Table 1 (Patient 4)
16	5	Jiao et al., 2022, Developmental Medicine & Child Neurology	Table 1 (Patient 5)
17	6	Jiao et al., 2022, Developmental Medicine & Child Neurology	Table 1 (Patient 6)
18	9	Jiao et al., 2022, Developmental Medicine & Child Neurology	Table 1 (Patient 9)
19	indvSG	Hamdan et al., 2017, American Journal of Human Genetics	Table 5 (indvSG)
20	HSJ0762	Hamdan et al., 2017, American Journal of Human Genetics	Table 5 (HSJ0762)
21	6	Sedlackova et al., 2024, European Journal of Paediatric Neurology	Table 2 (Patient 6)
22	1197	Fernández-Marmiesse et al., 2019, Frontiers in Neuroscience	Supplementary Table (Case 1197)
23	Case study 2	Josephs et al., 2019, Clinical Medicine	Case report

:::
